# Supplementary material for: Safety and Efficacy of Bispecific Antibodies in Adults with Large B-Cell Lymphomas: A Systematic Review of Clinical Trial Data
Source: Int J Mol Sci. 2024 Sep 9;25(17):9736. doi: 10.3390/ijms25179736 (PMC11396745; doi:10.3390/ijms25179736)
Supplement: Supplementary file 1 [file ijms-25-09736-s001.zip › Bayly-McCready_LBCLBsAbs_IJMS_SupplementaryMaterialS1_SearchStrategy.pdf]

Table S1a. Database search history

Database	Search date	Controlled vocabulary	Limitations	Search statement(s)		Results	Included
MEDLINE (PubMed)	10 April 2024	Toxicity<br>Side effect<br>Adverse effect<br>Adverse event<br>Safety<br>Tolerability<br>Morbidity<br>Mortality<br>Risk<br>Lymphoma<br>B-cell lymphoma<br>DLBCL<br>B-Cell NHL<br>LBCL<br>Transformed lymphoma<br>Malignant transformation<br>Richters<br>Bispecific antibody<br>Trispecific antibody<br>T-cell engager<br>Duobody<br>Bifunctional antibody<br>BsAb<br>BsMAb	English language<br><br>Published 2008 until current<br><br>No filters	1	toxicit* OR "side effect*" OR "adverse effect*" OR "adverse event*" OR safety OR tolerabilit* OR morbidit* OR mortalit* OR risk	7,360,833	6
				2	lymphoma* OR DLBCL OR "B-cell NHL" OR LBCL OR "transformed lymphoma" OR "malignant transformation" OR richter*	355,474	
				3	"bispecific antibody"[title/abstract:~3] OR "trispecific antibody"[title/abstract:~3] OR "t-cell engager"[title/abstract:~3] OR diabod* OR duobod* OR "bifunctional antibod*" OR BsAb* OR BsMAb	5,165	
				4	#1 AND #2 AND #3	342	
				5	Filters applied: English, from 2008/1/1 - 3000/12/12.	**287**	

Table S1a. *cont.*

Database	Search date	Controlled vocabulary	Limitations	Search statement(s)		Results	Included
EMBASE (OVID)	10 April 2024	Toxicity<br>Side effect<br>Adverse effect<br>Adverse event<br>Safety<br>Tolerability<br>Morbidity<br>Mortality<br>Risk<br>Lymphoma<br>B-cell lymphoma<br>DLBCL<br>B-Cell NHL<br>LBCL<br>Transformed lymphoma<br>Malignant transformation<br>Richters<br>Bispecific antibody<br>Trispecific antibody<br>T-cell engager<br>Duobody<br>Bifunctional antibody<br>BsAb<br>BsMAb	English language<br><br>Published 2008 until current<br><br>No filters	1	exp toxicity/ or toxicit*.mp.	1,676,640	12
				2	exp side effect/ or side effect*.mp.	1645,602	
				3	adverse effect*.mp.	318,617	
				4	exp adverse event/ or adverse event*.mp.	1,334,264	
				5	safety.mp. or safety/	1,548,969	
				6	exp tolerability/ or tolerability.mp.	230,229	
				7	exp morbidity/ or morbidit*.mp.	900,298	
				8	exp mortality/ or mortalit*.mp.	2,071,125	
				9	exp risk/ or risk.mp.	5,217,111	
				10	exp lymphoma/ or lymphoma*.ti,ab,kf.	455,002	
				11	(b-cell adj3 lymphoma).ti,ab,kf.	70,759	
				12	DLBCL.mp.	26,326	
				13	b-cell NHL.mp.	2,083	
				14	LBCL.mp.	1,152	
				15	(transformed adj3 lymphoma).ti,ab,kf.	1,380	
				16	malignant transform*.mp. or exp malignant transformation/	58,378	
				17	exp Richter syndrome/ or richter*.mp.	7,353	
				18	(bispecific adj3 antibod*).ti,ab,kf. or *bispecific antibody/	8,712	
				19	(trispecific adj3 antibod*).ti,ab,kf. or *trispecific antibody/	142	
				20	(t-cell adj3 engag*).mp.	4,304	
				21	diabod*.mp.	676	
				22	duobod*.mp.	81	
				23	bifunctional antibod*.mp.	207	
				24	BsAb*.mp.	1,468	
				25	BsMAb*.mp.	119	
				26	1 or 2 or 3 or 4 or 5 or 6 or 7 or 8 or 9	10,158,432	
				27	10 or 11 or 12 or 13 or 14 or 15 or 16 or 17	515,147	
				28	18 or 19 or 20 or 21 or 22 or 23 or 24 or 25	12,804	
				29	26 and 27 and 28	1,309	
				30	limit 29 to english language	1,289	
				31	limit 30 to yr="2008 -Current"	**1,233**	

Table S1a. *Cont.*

Database	Search date	Controlled vocabulary	Limitations	Search statement(s)		Results	Included
Cochrane Central Register of Controlled Trials (CENTRAL)	10 April 2024	Toxicity<br>Side effect<br>Adverse effect<br>Adverse event<br>Safety<br>Tolerability<br>Morbidity<br>Mortality<br>Risk<br>Lymphoma<br>B-cell lymphoma<br>DLBCL<br>B-Cell NHL<br>LBCL<br>Transformed lymphoma<br>Malignant transformation<br>Richters<br>Bispecific antibody<br>Trispecific antibody<br>T-cell engager<br>Duobody<br>Bifunctional antibody<br>BsAb<br>BsMAb	Published 2008 until current<br><br>No filters	1	toxicit*	57,176	0
				2	side effect*	186,226	
				3	adverse effect*	338,726	
				4	adverse event*	181,258	
				5	Safety	321,498	
				6	tolerabilit*	82,609	
				7	morbidit*	54,064	
				8	mortalit*	125,491	
				9	Risk	310,627	
				10	lymphoma*	15,529	
				11	B-cell NEAR/3 lymphoma	3,119	
				12	DLBCL	1,373	
				13	B-cell NHL	678	
				14	LBCL	85	
				15	transformed lymphoma*	315	
				16	malignant transformation	460	
				17	Richter*	2,404	
				18	bispecific NEAR/3 antibod*	366	
				19	trispecific NEAR/3 antibod*	1	
				20	t-cell NEAR/3 engag*	71	
				21	diabod*	4	
				22	duobod*	4	
				23	bifunctional antibod*	28	
				24	BsAb*	53	
				25	BsMAb*	3	
				26	{OR #1-#9}	895,337	
				27	{OR #10-#17}	18,243	
				28	{OR #18-#25}	496	
				29	#26 AND #27 AND #28	81	
				30	#29 with Cochrane Library publication date from Jan 2008 to present, in Trials	**76**	

Table S1a. *Cont.*

Database	Search date	Controlled vocabulary	Limitations	Search statement(s)		Results		Results after screening
medRxiv and bioRxiv	10 April 2024	Lymphoma<br>B-cell<br>DLBCL<br>LBCL<br>Transformed lymphoma<br>Malignant transformation<br>Richters<br>Bispecific antibody<br>Trispecific antibody<br>T-cell engager<br>Duobody<br>Bifunctional antibody	Published 2008 until current<br><br>No filters	All search statements performed in "Abstract or Title" field (match all words), with limit "posted between 01-Jan-2008 –" applied		medRxiv	bioRxiv	0
				1	lymphoma* bispecific	2	7	
				2	b-cell bispecific	1	9	
				3	DLBCL bispecific	0	2	
				4	LBCL bispecific	0	0	
				5	transformed lymphoma* bispecific	0	0	
				6	malignant transform* bispecific	0	0	
				7	richter* bispecific	0	0	
				8	lymphoma* trispecific	0	0	
				9	b-cell trispecific	0	0	
				10	DLBCL trispecific	0	0	
				11	LBCL trispecific	0	0	
				12	transformed lymphoma* trispecific	0	0	
				13	malignant transform* trispecific	0	0	
				14	richter* trispecific	0	0	
				15	lymphoma* t-cell engag*	0	0	
				16	b-cell t-cell engag*	0	0	
				17	DLBCL t-cell engag*	0	0	
				18	LBCL t-cell engag*	0	0	
				19	transformed lymphoma* t-cell engag*	0	0	
				20	malignant transform* t-cell engag*	0	0	
				21	richter* t-cell engag*	0	0	
				22	lymphoma* duobod*	0	0	
				23	b-cell duobod*	0	0	
				24	DLBCL duobod*	0	0	
				25	LBCL duobod*	0	0	
				26	transformed lymphoma* duobod*	0	0	
				27	malignant transform* duobod*	0	0	
				28	richter* duobod*	0	0	
				29	lymphoma* bifunctional antibod*	0	0	
				30	b-cell bifunctional antibod*	0	0	
				31	DLBCL bifunctional antibod*	0	0	
				32	LBCL bifunctional antibod*	0	0	
				33	transformed lymphoma* bifunctional antibod*	0	0	
				34	malignant transform* bifunctional antibod*	0	0	

				35	richter* bifunctional antibod*	0	0	
				Total from all searches		**3**	**18**	

Table S1b. Grey literature search results

Location	Date of search	Search limits	Search focus	Number of papers selected for screening	Results after screening
American Society of Clinical Oncology (ASCO) conference abstracts	11 April 2024	Filters: abstracts (media type)	Keyword search:<br>• bispecific antibody<br><br>Within topics:<br>1. Cancers (101)<br>a. haematological malignancies (33)<br>i. lymphoma (16)<br>2. Treatment (80)<br>a. Outcomes (66)<br>i. Treatment related complications (25)<br>ii. Mortality (1)<br>b. Drug therapy (19)<br>i. antibody therapy (7)<br>c. Immunotherapy (6)<br>i. antibody therapy (1)<br>3. Research (70)<br>a. Clinical research (66)<br>i. clinical trials (66)<br>b. Research studies (7)<br>i. Escalation studies (5)<br>ii. Validation studies (2)	4	0
American Association for Cancer Research (AACR) conference abstracts	15 April 2024	Selected conferences – abstract books 2008-2024	• Hematologic Malignancies: Translating Discoveries to Novel Therapies (2017)<br>• AACR Special Conference: Tumor Immunology and Immunotherapy (2018 - 2023)<br>• AACR-NCI-EORTC International Conference on Molecular Targets and Cancer Therapeutics (2019, 2023)<br>• AACR Annual Meeting (2008 – 2024)<br>• Blood Cancer Discovery Symposium (2024)	0	Not applicable

Table S1b. *Cont.*

Location	Date of search	Search limits	Search focus	Number of papers selected for screening	Results after screening
American Society of Hematology (ASH) conference abstracts	12 April 2024	Published 2008 until 2024	Keyword search:<br>• bispecific antibody<br>• lymphoma<br>• safety<br>• toxicity	62	0
European Hematology Association (EHA) conference abstracts	13 April 2024	Abstract books (all years)	Within topics:<br>• Aggressive lymphoma – Novel Agents<br>• Aggressive lymphoma – Clinical<br>• Aggressive non-Hodgkin lymphoma – Clinical<br>• Aggressive lymphoma – Clinical/non CAR T<br>• Aggressive lymphomas – combination therapies<br>• Aggressive lymphomas – prospective studies<br>• Aggressive lymphomas: Cellular and bispecific antibody therapies<br>• Aggressive B-NHL: Immunotherapy<br>• Diffuse large b-cell lymphoma<br>• Aggressive lymphoma with emphasis on novel agents	19	0
Lugano International Conference on Malignant Lymphoma (ICML) conference abstracts	14 April 2024	10-ICML to 17-ICML abstracts (2008-2023)	All abstracts	20	1
Citation mining	25 April 2024	Not applicable	Reference lists of included studies	1	0
