# Supplementary material for: Safety and Efficacy of Bispecific Antibodies in Adults with Large B-Cell Lymphomas: A Systematic Review of Clinical Trial Data
Source: Int J Mol Sci. 2024 Sep 9;25(17):9736. doi: 10.3390/ijms25179736 (PMC11396745; doi:10.3390/ijms25179736)
Supplement: Supplementary file 1 [file ijms-25-09736-s001.zip › Bayly-McCredie_LBCLBsAbs_IJMS_SupplementaryMaterialS2_Intervention_AdverseEvent_Characteristics.pdf]

Table S2. Intervention and adverse event study characteristics

Citation	Treatment schedule	Median exposure	Adverse event mitigation strategies	Adverse event criteria
Viardot et al., 2016[29]	Continuous IV infusion with step-up dosing up to 112/micrograms/day for up to 8 weeks (cycle 1), followed by 4 treatment-free weeks<br><br>Patients with SD or better could receive 4 weeks consolidation (cycle 2)	46.8 days (IQR 22.1-76.9)	Mandatory inpatient monitoring for at least 3 days after start of infusion or increase in dose<br><br>Premedication with steroids	All adverse events graded using NCI CTCAE v4.0
Gaballa et al., 2023[30]	AZD0486 was given via IV infusion with either no step-up dose on day 1 and day 15 at 0.03–2.4mg, 1 step-up dose on day 1 at 0.27–1mg and day 15 at 2.4–10mg, or 2 step-up on day 1 at 0.27mg, day 8 at 1mg, and day 15 at 2.4–7.2mg schedules<br><br>Treatment was given Q2W in 28-day cycles up to 2 years, or Q4W for patients in CR at cycle 6	Not reported	Not reported	CRS and ICANS graded according to ASTCT consensus grading, 2019<br><br>All other adverse events graded according to NCI CTCAE v5.0
Hawkes et al., 2022[31]	TG-1801 and ublituximab were administered via IV infusion in 28-day cycles<br><br>TG-1801 was administered at 300mg or 400mg with 900mg of ublituximab on day 1, 8, and 15 of cycle 1, day 1 of cycle 2–6, and day 1 of cycles 9–24	8.7 months, (range 1-20 months)	CRS/ICANS mitigation strategies not required*<br><br>No other adverse event mitigation strategies reported	CRS/ICANS grading not required*<br><br>All other adverse events graded according to NCI CTCAE v5.0*
Bannerji et al., 2022[32]	Odronextamab was administered via IV infusion with step-up doses during cycle 1 from 0.1mg to the target dose of 320mg<br><br>QW dosing during cycles 2–4, Q2W from cycle 5 continuous until disease progression	10 doses (IQR 4–20), 13.1 weeks (IQR 5.0–32.0)	Mandatory inpatient monitoring for approximately 24-hours after the end of each infusion up to cycle 2 day 1<br><br>Premedication up to cycle 2 day 8 with steroids, paracetamol, and diphenhydramine<br><br>Local institutional practice for prescribing of anti-infective prophylaxis	CRS graded according to modified Lee et al, 2014 or ASTCT consensus grading, 2019 depending on the patient enrolment date<br><br>A sponsor-defined list of neurological adverse events consistent with ICANS was used<br><br>All other adverse events graded according to NCI CTCAE v4.03

Table S2. *Cont.*

Citation	Treatment Schedule	Median exposure	Adverse event mitigation strategies	Adverse event criteria
Melchardt et al., 2023[33]	Pre-phase steroids<br>Cycle 1 (21 days) consisted of IV obinutuzumab 1000mg on day 1, IV polatuzumab vedotin 1.8mg/kg on day 2, IV glofitamab 2.5mg on day 8 and 10mg on day 15<br><br>Cycles 2–6 consisted of IV rituximab 375mg/m2 and polatuzumab vedotin 1.8mg/kg on day 1, and glofitamab 30mg on day 2<br><br>Glofitamab 30mg continued on day 1 of cycles 7–12	Not reported	Not reported	Not reported
Hutchings et al., 2019[34]	Pretreatment with obinutuzumab (1000mg IV) was administered on day -7 of cycle 1<br><br>Glofitamab was administered IV Q3W from cycle 1 day 1<br><br>Atezolizumab 1200mg was given with glofitamab from cycle 2 onwards	Not reported	CRS risk mitigation with pretreatment obinutuzumab	CRS according to Lee at al, 2014<br><br>Criteria used for all other adverse events was not reported
Hutchings et al., 2023[35]	Pretreatment with obinutuzumab (1000 mg) was administered 7 days prior to glofitamab<br><br>Glofitamab was then administered IV as step-up doses on day 8 (2.5 mg) and day 15 (10 mg) of cycle 1, followed by 30 mg on day 1 of cycles 2 through 12 in 21-day cycles<br><br>RO7227166 was given IV on cycle 2 day 8, then day 1 of cycles 2–12 with glofitamab	Not reported	CRS risk mitigation with pretreatment obinutuzumab	CRS/ICANS grading not reported<br><br>All other adverse events graded according to NCI CTCAE v5.0**

Table S2. *Cont.*

Citation	Treatment Schedule	Median exposure	Adverse event mitigation strategies	Adverse event criteria
Dickinson et al., 2022[36]	Glofitamab was administered via IV infusion as step-up doses on day 8 (2.5mg) and day 15 (10mg) of cycle 1, followed by 30mg on day 1 of cycles 2–12<br><br>Pretreatment with obinutuzumab (1000mg) was administered 7 days prior to glofitamab	79 days (range 1–326 days), 5 cycles (range 1–13 cycles)	Mandatory inpatient monitoring for first dose, then optional unless they had a history of CRS<br><br>CRS risk mitigation with pretreatment obinutuzumab<br><br>Premedication with steroids, paracetamol and antihistamine prior to each dose<br><br>Anti-infective prophylaxis as per institutional practice	CRS graded according to Lee et al, 2014<br><br>ICANS events were reported using NCI CTCAE neurological adverse events consistent with ICANS<br><br>All other adverse events graded according to NCI CTCAE v4.03
Falchi et al., 2023[37]	Subcutaneous epcoritamab QW in cycles 1–4 and Q3W in cycles 5–6, followed by Q4W in cycles 7–12<br><br>R-CHOP was given during cycles 1–6	Median relative dose intensity of ≥94%	Not reported	Not reported
Vermaat et al., 2023[38]	Subcutaneous epcoritamab was administered QW for cycles 1–2 (21 days each), Q3W for cycles 3–6 (21 days each), and Q4W for cycles 7–8 (28 days each) with R-mini-CHOP Q3W cycles 1–6	Not reported	Not reported	Not reported
Brody et al., 2023[39]	Subcutaneous epcoritamab was administered with step-up dosing on cycle 1 day 1 and cycle 1 day 8, followed by 48mg QW for cycle 1 day 15 to cycle 3 day 22, Q2W for cycles 4–9, and Q4W for cycles ≥10 onwards until withdrawal/progressive disease<br><br>GemOx was administered Q2W in cycles 1–4	Not reported	Corticosteroid prophylaxis during cycle 1	Not reported

Table S2. *Cont.*

Citation	Treatment Schedule	Median exposure	Adverse event mitigation strategies	Adverse event criteria
Abrisqueta et al., 2022[40]	Standard R-DHAX/C and subcutaneous epcoritamab (21-day cycles: QW, cycles 1–3)<br><br>If HDT-ASCT was deferred, patients could continue epcoritamab monotherapy (21-day cycle: QW, cycle 4; 28-day cycles: Q2W, cycles 5–9, and Q4W, cycles ≥10) until disease progression or withdrawal	Not reported	Corticosteroid prophylaxis during cycle 1	Not reported
Thieblemont et al., 2023[41]	Epcoritamab was administered subcutaneously at 0.16mg on cycle 1 day 1, 0.8-mg on cycle 1 day 8 and then 48mg weekly from cycle 1 day 15 to cycle 3 day 22, Q2W from cycles 4–9 and then Q4W from cycle 10 onwards until disease progression	5 cycles (15 doses), (range, 1–20 cycles)	Mandatory inpatient monitoring for 24-hours following the first full dose of 48mg<br><br>Premedication with steroids, antihistamine and paracetamol during cycle 1, optional for later cycles<br><br>Anti-infective prophylaxis recommended for most patients, mandatory in high-risk patients	CRS and ICANS graded according to ASTCT consensus grading, 2019<br><br>All other adverse events graded according to NCI CTCAE v5.0
Vose et al., 2023[42]	Subcutaneous epcoritamab was administered at 0.16-mg on cycle 1 day 1, 0.8-mg on cycle 1 day 8 and then 48mg weekly from cycle 1 day 15 to cycle 3 day 22, Q2W from cycles 4 to 9 and then Q4W from cycle 10 onwards until disease progression	Not reported	Hospitalization and inpatient monitoring were optional<br><br>Premedication with 15mg dexamethasone, diphenhydramine and paracetamol during cycle 1<br><br>Dexamethasone as CRS prophylaxis on days 2-4, 9-11, 16-18, and 23-25 of cycle 1<br><br>Patients were recommended to withhold antihypertensives 24-hours prior to dosing, to be well hydrated, and monitor their temperature regularly during cycle 1	CRS and ICANS graded according to ASTCT Consensus Grading, 2019*<br><br>All other adverse events graded according to NCI CTCAE v5.0*

Table S2. *Cont.*

Citation	Treatment Schedule	Median exposure	Adverse event mitigation strategies	Adverse event Criteria
Patel et al., 2022[43]	Plamotamab was administered via IV infusion for up to 8 doses at the following doses: 0.8mg on cycle 1 day 1, 2mg on cycle 1 day 8, 20mg on cycle 1 day 15, 35mg on cycle 1 day 22, and 50mg on cycle 2 day 1, cycle 2 day 8, cycle 2 day 15, and cycle 2 day 22	Not reported	Mandatory inpatient monitoring for 24-hours following the first full dose of 48mg<br><br>Premedication with steroids, paracetamol and antihistamine[82]<br><br>Anti-infective prophylaxis not reported	CRS and ICANS grading not reported<br><br>All other adverse events graded according to NCI CTCAE v4.03**
Song et al., 2023[44]	GB261 was administered via IV infusion at flat or step-up doses ranging from 1mg to 300mg administered weekly in 21-day cycles continuously until disease progression	Not reported	Not reported	CRS graded according to ASTCT consensus grading, 2019<br><br>Criteria for grading all other adverse events was not reported
Olszewski et al., 2023[45]	Mosunetuzumab was administered via IV infusion with 1mg on cycle 1 day 1, 2mg on cycle 1 day 8, 60mg on cycle 1 day 15 and 30mg Q3W from cycle 2 day 1 onwards for up to 8 cycles (for patients achieving CR), or 17 cycles (for patients with SD or PR)<br><br>CHOP was given with mosunetuzumab for 6 cycles	8 doses (range 1–13 doses)	Premedication with steroids, paracetamol and antihistamine<br><br>Prophylaxis with G-CSF during the 6 cycles of mosunetuzumab-CHOP was mandatory<br><br>Anti-infection prophylaxis was optional	CRS graded according to ASTCT consensus grading, 2019<br><br>ICANS graded according to NCI CTCAE v5.0, then described as "potentially consistent with ICANS" based on type and timing*<br><br>All other adverse events graded according to NCI CTCAE v5.0

Supplementary Material S2. *Cont.*

Citation	Treatment Schedule	Median exposure	Adverse event mitigation strategies	Adverse event criteria
Matasar et al., 2024[46]	Mosunetuzumab was administered via IV infusion with 1mg on cycle 1 day 1, 2mg on cycle 1 day 8, 60mg on cycle 1 day 15 and cycle 2 day 1<br><br>Treatment continued at 30mg Q3W from cycle 3 onwards for up to 8 cycles (for patients achieving CR), or 17 cycles (for patients with SD or PR after 8 cycles)	8 cycles (range 1–17 cycles)	Optional inpatient monitoring<br><br>Premedication with steroids during cycles 1–2, then optional<br><br>Additional premedication with antipyretics and/or antihistamines was optional<br><br>Anti-infective prophylaxis as per institutional practice	CRS graded according to ASTCT consensus grading, 2019<br><br>All other adverse events graded according to NCI CTCAE v4.0[83]
Budde et al., 2024[47]	Mosunetuzumab was administered via IV infusion with 1mg on cycle 1 day 1, 2mg on cycle 1 day 8, 60mg on cycle 1 day 15 and 30mg Q3W from cycle 2 day 1 onwards for up to 8 cycles (for patients achieving CR), or 17 cycles (for patients with SD or PR)<br><br>Polatuzumab vedotin was given with mosunetuzumab for 6 cycles	4.9 months (8 cycles)	Optional inpatient monitoring<br><br>Premedication with steroids during cycles 1–2, then optional<br><br>Additional premedication with antipyretics and/or antihistamines was optional<br><br>Anti-infective prophylaxis as per institutional practice	CRS graded according to ASTCT consensus grading, 2019<br><br>All other adverse events graded according to NCI CTCAE v5.0

*Personal communication with author. **Data taken from ClinicalTrials.gov.

Abbreviations: IV, intravenous; SD, stable disease; IQR, interquartile range; NCI CTCAE, National Cancer Institute Common Terminology Criteria for Adverse Events; v, version; mg, milligrams; Q2W, every 2 weeks; Q4W, every 4 weeks; CR, complete response; CRS, cytokine release syndrome; ICANS, immune effector cell-associated neurotoxicity syndrome; ASTCT, American Society for Transplantation and Cellular Therapy; QW, every week; Q3W, every 3 weeks; R-CHOP, rituximab/cyclophosphamide/doxorubicin/vincristine/prednisolone; GemOx, gemcitabine/oxaliplatin; R-DHAX/C, rituximab/dexamethasone/cytarabine/and oxaliplatin or carboplatin; HDT, high-dose therapy; ASCT, autologous stem-cell transplant; PR, partial response; G-CSF, granulocyte-colony stimulating factor.

References

29. Viardot A, Goebeler ME, Hess G, Neumann S, Pfreundschuh M, Adrian N, et al. Phase 2 study of the bispecific T-cell engager (BiTE) antibody blinatumomab in relapsed/refractory diffuse large B-cell lymphoma. Blood. 2016;127(11):1410-6.
30. Gaballa S, Nair R, Jacobs RW, Devata S, Cho SG, Stevens DA, et al. Double step-up dosing (2SUD) regimen mitigates severe ICANS and CRS while maintaining high efficacy in subjects with relapsed/refractory (R/R) B-cell non-Hodgkin lymphoma (NHL) treated with AZD0486, a novel CD19xCD3 T-cell engager (TCE): Updated safety and efficacy data from the ongoing first-in-human (FIH) phase 1 trial. Blood. 2023;142(Supplement 1):1662.
31. Hawkes E, Lewis KL, Wong Doo N, Patil SS, Miskin HP, Sportelli P, et al. First-in-human (FIH) study of the fully-human kappa-lambda CD19/CD47 bispecific antibody TG-1801 in patients (pts) with B-cell lymphoma. Blood. 2022;140(Supplement 1):6599-601.
32. Bannerji R, Arnason JE, Advani RH, Brown JR, Allan JN, Ansell SM, et al. Odronextamab, a human CD20×CD3 bispecific antibody in patients with CD20-positive B-cell malignancies (ELM-1): results from the relapsed or refractory non-Hodgkin lymphoma cohort in a single-arm, multicentre, phase 1 trial. Lancet Haematology. 2022;9(5):e327-e39.
33. Melchardt T, Wurm-Kuczera RI, Altmann B, Pichler P, Orlinger M, Panny M, et al. Feasibility and safety of the first-in-human chemotherapy-light combination of rituximab, polatuzumab vedotin and glofitamab in previously untreated aggressive B-cell lymphoma patients above 60 uears of age ineligible for a fully dosed R-CHOP - R-Pola-Glo/Ikf-t062, a study of the Austrian Group for Medical Tumor Therapy (AGMT-NHL-16) and the German Lymphoma Alliance (GLA2022-10). Blood. 2023;142(Supplement 1):1734.
34. Hutchings M, Gritti G, Sureda A, Terol MJ, Dyer MJS, Iacoboni G, et al. CD20-TCB, a novel T-cell-engaging bispecific antibody, can be safely combined with the anti-PD-L1 antibody atezolizumab in relapsed or refractory B-cell non-hodgkin lymphoma. Blood Conference: 61st Annual Meeting of the American Society of Hematology, ASH. 2019;134(Supplement 1).
35. Hutchings M, Dickinson M, Carlo-Stella C, Morschhauser F, Bosch F, Gritti G, et al. Combining CD19-4-1BBL (RO7227166) with glofitamab is safe and shows early efficacy in patients suffering from relapsed or refractory B-cell non-Hodgkin lymphoma. Hematological Oncology. 2023;41(S2):136-8.
36. Dickinson MJ, Carlo-Stella C, Morschhauser F, Bachy E, Corradini P, Iacoboni G, et al. Glofitamab for relapsed or refractory diffuse large B-cell lymphoma. New England Journal of Medicine. 2022;387(24):2220-31.
37. Falchi L, Clausen MR, Offner F, de Vos S, Brody J, Linton KM, et al. Epcoritamab + R-CHOP in patients with previously untreated (1L) high-risk diffuse large B-cell lymphoma, including double-hit/ triple-hit lymphoma: Updated EPCORE NHL-2 data. Clinical Lymphoma, Myeloma and Leukemia. 2023;23(Supplement 1):S431-S2.
38. Vermaat JSP, Brody J, Duras J, Karimi YH, Cheah CY, Darrah JM, et al. Epcoritamab SC + R-Mini-CHOP leads to high complete metabolic response rates in patients with previously untreated diffuse large B-cell lymphoma ineligible for full-dose R-CHOP: First disclosure from arm 8 of the EPCORE NHL-2 trial. Blood. 2023;142(Supplement 1):4457.
39. Brody J, Joergensen JM, Belada D, Costello RT, Trneny M, Vitolo U, et al. Epcoritamab SC + GemOx leads to high complete metabolic response rates in patients with relapsed/refractory diffuse large B-cell lymphoma ineligible for autologous stem cell transplant: Updated results from Epcore NHL-2. Blood. 2023;142(Supplement 1):3092.
40. Abrisqueta P, Falchi L, Phillips TJ, De Vos S, Nijland M, Offner F, et al. Subcutaneous epcoritamab + R-DHAX/C in patients (pts) with relapsed or refractory (R/R) diffuse large B-cell lymphoma (DLBCL) eligible for autologous stem cell transplant (ASCT): Preliminary phase 1/2 results. Journal of Clinical Oncology Conference: Annual Meeting of the American Society of Clinical Oncology, ASCO. 2022;40(16 Supplement 1).
41. Thieblemont C, Phillips T, Ghesquieres H, Cheah CY, Clausen MR, Cunningham D, et al. Epcoritamab, a novel, subcutaneous CD3xCD20 bispecific T-cell-engaging antibody, in relapsed or refractory large B-cell lymphoma: Dose expansion in a phase I/II trial. Journal of Clinical Oncology. 2023;41(12):2238-47.
42. Vose JM, Feldman T, Chamuleau MED, Kim WS, Lugtenburg P, Kim TM, et al. Mitigating the risk of cytokine release syndrome (CRS): Preliminary results from a DLBCL cohort of Epcore NHL-1. Blood. 2023;142(Supplement 1):1729.

43. Patel K, Riedell PA, Tilly H, Ahmed S, Michot JM, Ghesquieres H, et al. A phase 1 study of plamotamab, an anti-CD20 x anti-CD3 bispecific antibody, in patients with relapsed/refractory non-Hodgkin's lymphoma: Recommended dose safety/efficacy update and escalation exposure-response analysis. Blood. 2022;140(Supplement 1):9470-2.
82. Patel K, Michot JM, Chanan-Khan AA, Salles GA, Cartron G, Peyrade F, et al. Preliminary safety and anti-tumor activity of XmAb13676, an anti-CD20 x anti-CD3 bispecific antibody, in patients with relapsed/refractory non-Hodgkin's lymphoma and chronic lymphocytic leukemia. Blood. 2019;134(Supplement 1):4079.
44. Song Y, Li L, Qian Z, Zhou K, Fan L, Tan P, et al. GB261, an Fc-function enabled and CD3 affinity de-tuned CD20/CD3 bispecific antibody, demonstrated a highly advantageous safety/efficacy balance in an ongoing first-in-human dose-escalation study in patients with relapsed/refractory non-Hodgkin lymphoma. Blood. 2023;142(Supplement 1):1719.
45. Olszewski AJ, Phillips TJ, Hoffmann MS, Armand P, Kim TM, Yoon DH, et al. Mosunetuzumab in combination with CHOP in previously untreated DLBCL: safety and efficacy results from a phase 2 study. Blood Advances. 2023;7(20):6055-65.
46. Matasar M, Bartlett NL, Shadman M, Budde LE, Flinn I, Gregory GP, et al. Mosunetuzumab safety profile in patients with relapsed/refractory B-cell non-Hodgkin lymphoma: Clinical management experience from a pivotal phase I/II trial. Clinical Lymphoma, Myeloma & Leukemia. 2024;24(4):240-53.
83. Budde LE, Assouline S, Sehn LH, Schuster SJ, Yoon S-S, Yoon DH, et al. Durable responses with mosunetuzumab in relapsed/refractory indolent and aggressive B-cell non-Hodgkin lymphomas: Extended follow-up of a phase I/II study. Journal of Clinical Oncology. 2024;42(19):2250-6.
47. Budde LE, Olszewski AJ, Assouline S, Lossos IS, Diefenbach C, Kamdar M, et al. Mosunetuzumab with polatuzumab vedotin in relapsed or refractory aggressive large B cell lymphoma: a phase 1b/2 trial. Nature Medicine. 2024;30(1):229-39.
