# Supplementary material for: Safety and Efficacy of Bispecific Antibodies in Adults with Large B-Cell Lymphomas: A Systematic Review of Clinical Trial Data
Source: Int J Mol Sci. 2024 Sep 9;25(17):9736. doi: 10.3390/ijms25179736 (PMC11396745; doi:10.3390/ijms25179736)
Supplement: Supplementary file 1 [file ijms-25-09736-s001.zip › Bayly-McCredie_LBCLBsAbs_IJMS_SupplementaryMaterialS3_FullSafetyEfficacy.pdf]

Table S3a. Reported rates of adverse events of special interest

Citation	CRS, %		ICANS, %		Infection, %		Fever/pyrexia, %		Fatigue, %	
	Any grade	Grade ≥3	Any grade	Grade ≥3	Any grade	Grade ≥3	Any grade	Grade ≥3	Any grade	Grade ≥3
Viardot et al., 2016[29]	0	0	NR	NR	NR	NR	43.5	4.3	26.1	0
Gaballa et al., 2023[30]	48	0	27	9.7	NR	NR	NR	NR	NR	NR
Hawkes et al., 2022[31, 51]	0	0	0	0	NR	NR	NR	NR	25	0
Bannerji et al., 2022[32]	61	7	12	3	49	23	73	1	33	5
Melchardt et al., 2023[33]	30	0	0	0	60	20	NR	NR	NR	NR
Hutchings et al., 2019[34]	42	0	NR	NR	NR	NR	37	NR	24	NR
Hutchings et al., 2023[35]	58.3	1.3	NR	NR	NR	NR	16.7	NR	17.9	NR
Dickinson et al., 2022[36]	63	3.9	8	3	38	15	18.2	0	11.7	0.6
Falchi et al., 2023[37]	60	2	2.1	0	NR	NR	40	NR	40	NR
Vermaat et al., 2023[38]	43	4	0	0	NR	NR	NR	NR	25	NR
Brody et al., 2023[39]	56	3	2.9	0	NR	NR	NR	NR	NR	NR
Abrisqueta et al., 2022[40]	41	0	3.4	0	NR	NR	NR	NR	28	NR
Thieblemont et al., 2023[41]	49.7	2.5	6.4	0.6	45.2	14.6	23.6	0	22.9	1.9
Vose et al., 2023[42]	17	0	0	0	38	NR	NR	NR	21	NR
Patel et al., 2022[43]	72.2	0	NR	NR	NR	NR	38.9	NR	NR	NR
Song et al., 2023[44]	12.8	0	0	0	NR	NR	NR	NR	NR	NR
Olszewski et al., 2023[45]	60	0	12.5	2.5	52.5	22.5	20	0	47.5	10
Matasar et al., 2024[46]	39.4	2.8	0.9	0	46.8	15.1	24.3	1.8	32.1	0.9
Budde et al., 2024[47]	16.7	2.5	4.9	1.6	39.2	10.8	20	NR	46.7	6.7

Abbreviations: CRS, cytokine release syndrome; ICANS, immune effector cell-associated neurotoxicity syndrome; NR, not reported

Table S3b. Reported rates of cytopenias

Citation	Anemia, %		Thrombocytopenia, %		Neutropenia, %		Leukopenia, %		Lymphopenia, %	
	Any grade	Grade ≥3	Any grade	Grade ≥3	Any grade	Grade ≥3	Any grade	Grade ≥3	Any grade	Grade ≥3
Viardot et al., 2016[29]	4.4*	NR	21.7	17.4	4.4	NR	17.4	17.4	NR	NR
Gaballa et al., 2023[30]	34	0	NR	NR	32	NR	20.9	NR	29	NR
Hawkes et al., 2022[31, 51]	31	6	25	13	19	19	NR	NR	NR	NR
Bannerji et al., 2022[32]	38	25	28	14	25	19	19	9	22	19
Melchardt et al., 2023[33]	10	0	NR	NR	NR	NR	NR	NR	NR	NR
Hutchings et al., 2019[34]	29	13	NR	NR	21	18	NR	NR	NR	NR
Hutchings et al., 2023[35]	22.5	NR	NR	NR	28.6	NR	NR	NR	NR	NR
Dickinson et al., 2022[36]	31	6	25	8	38	27	NR	NR	NR	3.2
Falchi et al., 2023[37]	62	NR	NR	NR	64	NR	NR	NR	NR	NR
Vermaat et al., 2023[38]	21	NR	NR	NR	32	NR	NR	NR	NR	NR
Brody et al., 2023[39]	56	NR	68	NR	56	NR	NR	NR	NR	NR
Abrisqueta et al., 2022[40]	45	NR	69	NR	41	NR	NR	NR	NR	NR
Thieblemont et al., 2023[41]	17.8	10.2	13.4	5.7	21.7	14.6	NR	NR	NR	NR
Vose et al., 2023[42]	13	NR	13	NR	13	NR	NR	NR	NR	NR
Patel et al., 2022[43]	44.5	19.4	NR	11.1	NR	16.7	NR	NR	NR	NR
Song et al., 2023[44]	NR	NR	NR	NR	31.9	17	NR	NR	NR	NR
Olszewski et al., 2023[45]	42.5	30	25	17.5	70	65	10	7.5	12.5	10
Matasar et al., 2024[46]	15.1	8.3	11.5	6.9	27.5	24.3	NR	NR	NR	NR
Budde et al., 2024[47]	12.5	NR	3.3	NR	35	25	5	NR	4.2	NR

*Data taken from ClinicalTrials.gov Abbreviations: NR, not reported

Table S3c. Other frequently reported adverse events

Citation	Any grade, occurring in >20% of participants		Grade ≥3, occurring in >5% of participants	
	**Adverse event**	**Rate, %**	**Adverse event**	**Rate, %**
Viardot et al., 2016[29]	Tremor<br>Oedema<br>Device-related infection<br>Pneumonia<br>Diarrhea	47.8<br>26.1<br>21.7<br>21.7<br>21.7	Device-related infection<br>Pneumonia<br>C-reactive protein (CRP) increased<br>Encephalopathy<br>Aphasia<br>Hyperglycemia	13<br>13<br>13<br>8.7<br>8.7<br>8.7
Gaballa et al., 2023[30]	N/A	N/A	NR	NR
Hawkes et al., 2022[31, 51]	Headache<br>Abdominal pain<br>COVID-19	25<br>25<br>25	Fall	6
Bannerji et al., 2022[32]	Chills<br>Hypophosphatemia<br>C-reactive protein (CRP) increased<br>Cough<br>Hypotension<br>Headache<br>Nausea<br>Infusion-related reaction<br>Decreased appetite<br>Dyspnea<br>Blood creatinine increased<br>Tachycardia<br>Oedema peripheral	47<br>29<br>28<br>28<br>28<br>25<br>24<br>24<br>23<br>22<br>21<br>21<br>21	Hypophosphatemia<br>Aspartate transaminase increased<br>Pneumonia<br>Hypotension<br>Alanine transaminase increased<br>Hypoxia<br>Hyperglycemia	19<br>10<br>9<br>8<br>7<br>6<br>6
Melchardt et al., 2023[33]	N/A	N/A	Bleeding<br>Renal toxicity	10<br>10
Hutchings et al., 2019[34]	Diarrhea<br>Decreased appetite	21<br>21	NR	NR
Hutchings et al., 2023[35]	COVID-19	25	NR	NR
Dickinson et al., 2022[36]	N/A	N/A	Hypophosphatemia	5.8
Falchi et al., 2023[37]	NR	NR	NR	NR
Vermaat et al., 2023[38]	Constipation<br>Hypokalemia	21<br>21	NR	NR
Brody et al., 2023[39]	Diarrhea<br>COVID-19	59<br>32	NR	NR
Abrisqueta et al., 2022[40]	Nausea	34	NR	NR
Thieblemont et al., 2023[41]	Injection site reactions<br>Diarrhea	28<br>20.4	NR	NR

Vose et al., 2023[42]	N/A	N/A	NR	NR
Patel et al., 2022[43]	Nausea<br>Asthenia<br>Diarrhea<br>Hypophosphatemia<br>Aspartate transaminase increased	38.9<br>27.8<br>25<br>25<br>25	NR	NR
Song et al., 2023[44]	COVID-19 infection	40.4	COVID-19 infection	12.8
Olszewski et al., 2023[45]	Nausea<br>Decreased appetite<br>Constipation<br>Peripheral neuropathy<br>Diarrhea<br>Hypokalemia<br>Vomit<br>Alopecia<br>Headache<br>Dizziness<br>Hypotension	55<br>37.5<br>37.5<br>32.5<br>32.5<br>32.5<br>30<br>30<br>25<br>25<br>22.5	Febrile neutropenia<br>Pneumonia<br>Hypokalemia<br>Decreased appetite	20<br>7.5<br>7.5<br>7.5
Matasar et al., 2024[46]	Hypophosphatemia<br>Headache	22.5<br>20.2	Hypophosphatemia	14.7
Budde et al., 2024[47]	Diarrhea<br>Peripheral neuropathy<br>Nausea<br>Decreased appetite<br>Headache	30.8<br>30.8<br>30<br>22.5<br>21.7	N/A	N/A

Abbreviations: N/A, not applicable; NR, not reported

Table S3d. Reported efficacy outcomes

Citation	n (cohort)	MFU, months	ORR, %	CRR, %	PRR, %	mDoR, months	mPFS, months	Response criteria
				Front-line				
Melchardt et al., 2023[33]	NR	NR	NR	NR	NR	NR	NR	NR
Falchi et al., 2023[37]	n = 46	11.5	100	76	NR	Not reached	Not reached	Lugano
Vermaat et al., 2023[38]	n = 20	NR	100	85	NR	NR	NR	Lugano
Olszewski et al., 2023[45]	n = 40	32	95	90	5	Not reached	NR	Lugano
				Relapsed/refractory				
Viardot et al., 2016[29]	n = 20 (cohort I + III)	15	40	20	20	11.6	3.7	Cheson
Gaballa et al., 2023[30]	NR	NR	NR	NR	NR	NR	NR	NR
Hawkes et al., 2022[31]	n = 9 (DLBCL, combination arm)	NR	56	0	56	NR	NR	NR
Bannerji et al., 2022[32]	n = 49 (DLBCL, CAR T naïve)/ n = 33 (DLCBL, CAR T exposed)	4.2	39 / 33	24 / 24	14 / 9	4.4 / not reached	11.5 / 2	Lugano
Hutchings et al., 2019[34]	n = 31 (aNHL patients)	NR	29	9.7	NR	NR	NR	Lugano
Hutchings et al., 2023[35]	n = 57 (aNHL patients)	NR	70	53	NR	NR	NR	Lugano
Dickinson et al., 2022[36]	n = 155	12.6	52	39	NR	18.4	4.9	Lugano
Brody et al., 2023[39]	n = 34	NR	91	59	NR	NR	NR	Lugano
Abrisqueta et al., 2022[40]	n = 15 (received epcoritamab + R-DHAX/C)	9.2	100	80	20	Not reached	NR	Lugano
Thieblemont et al., 2023[41]	n = 157	10.7	63.1	38.9	24.2	12	4.4	Lugano
Vose et al., 2023[42]	NR	NR	NR	NR	NR	NR	NR	NR
Patel et al., 2022[43]	n = 19 (DLBCL patients)	NR	47.4	26.3	NR	NR	NR	NR
Song et al., 2023[44]	n = 22 (dosed at ≥3mg GB261)	4.5	73	45.5	NR	Not reached	NR	Lugano & LYRIC 2016

Matasar et al., 2024[46, 52]	n = 129	11.9	34.9	19.4	15.5	NR	NR	Cheson
Budde et al., 2024[47]	n = 98 (dose expansion)	23.9	59.2	45.9	NR	20.8	11.4	Lugano

Abbreviations: n, number (of participants); MFU, median follow-up; ORR, overall response rate; CRR, complete response rate; PRR, partial response rate; mDoR, median duration of response; mPFS, median progression free survival; NR, not reported; DLBCL, diffuse large B-cell lymphoma; CAR, chimeric antigen receptor; aNHL, aggressive non-Hodgkin's lymphoma; R-DHAX/C, rituximab/dexamethasone/cytarabine/oxaliplatin or carboplatin

References

29. Viardot A, Goebeler ME, Hess G, Neumann S, Pfreundschuh M, Adrian N, et al. Phase 2 study of the bispecific T-cell engager (BiTE) antibody blinatumomab in relapsed/refractory diffuse large B-cell lymphoma. Blood. 2016;127(11):1410-6.

30. Gaballa S, Nair R, Jacobs RW, Devata S, Cho SG, Stevens DA, et al. Double step-up dosing (2SUD) regimen mitigates severe ICANS and CRS while maintaining high efficacy in subjects with relapsed/refractory (R/R) B-cell non-Hodgkin lymphoma (NHL) treated with AZD0486, a novel CD19xCD3 T-cell engager (TCE): Updated safety and efficacy data from the ongoing first-in-human (FIH) phase 1 trial. Blood. 2023;142(Supplement 1):1662.

31. Hawkes E, Lewis KL, Wong Doo N, Patil SS, Miskin HP, Sportelli P, et al. First-in-human (FIH) study of the fully-human kappa-lambda CD19/CD47 bispecific antibody TG-1801 in patients (pts) with B-cell lymphoma. Blood. 2022;140(Supplement 1):6599-601.

51. Hawkes E, Lewis K, WongDoo N, Patil S, Miskin H, Sportelli P, et al. First-in-human (FIH) study of the fully-human kappa-lambda CD19/CD47 bispecific antibody TG-1801 in patients (pts) with B cell lymphoma: Poster session presented at: American Society of Hematology 64th Congress; 2022 Dec 11; New Orleans, LA.

32. Bannerji R, Arnason JE, Advani RH, Brown JR, Allan JN, Ansell SM, et al. Odronextamab, a human CD20×CD3 bispecific antibody in patients with CD20-positive B-cell malignancies (ELM-1): results from the relapsed or refractory non-Hodgkin lymphoma cohort in a single-arm, multicentre, phase 1 trial. Lancet Haematology. 2022;9(5):e327-e39.

33. Melchardt T, Wurm-Kuczera RI, Altmann B, Pichler P, Orlinger M, Panny M, et al. Feasibility and safety of the first-in-human chemotherapy-light combination of rituximab, polatuzumab vedotin and glofitamab in previously untreated aggressive B-cell lymphoma patients above 60 uears of age ineligible for a fully dosed R-CHOP - R-Pola-Glo/Ikf-t062, a study of the Austrian Group for Medical Tumor Therapy (AGMT-NHL-16) and the German Lymphoma Alliance (GLA2022-10). Blood. 2023;142(Supplement 1):1734.

34. Hutchings M, Gritti G, Sureda A, Terol MJ, Dyer MJS, Iacoboni G, et al. CD20-TCB, a novel T-cell-engaging bispecific antibody, can be safely combined with the anti-PD-L1 antibody atezolizumab in relapsed or refractory B-cell non-hodgkin lymphoma. Blood Conference: 61st Annual Meeting of the American Society of Hematology, ASH. 2019;134(Supplement 1).

35. Hutchings M, Dickinson M, Carlo-Stella C, Morschhauser F, Bosch F, Gritti G, et al. Combining CD19-4-1BBL (RO7227166) with glofitamab is safe and shows early efficacy in patients suffering from relapsed or refractory B-cell non-Hodgkin lymphoma. Hematological Oncology. 2023;41(S2):136-8.

36. Dickinson MJ, Carlo-Stella C, Morschhauser F, Bachy E, Corradini P, Iacoboni G, et al. Glofitamab for relapsed or refractory diffuse large B-cell lymphoma. New England Journal of Medicine. 2022;387(24):2220-31.

37. Falchi L, Clausen MR, Offner F, de Vos S, Brody J, Linton KM, et al. Epcoritamab + R-CHOP in patients with previously untreated (1L) high-risk diffuse large B-cell lymphoma, including double-hit/ triple-hit lymphoma: Updated EPCORE NHL-2 data. Clinical Lymphoma, Myeloma and Leukemia. 2023;23(Supplement 1):S431-S2.

38. Vermaat JSP, Brody J, Duras J, Karimi YH, Cheah CY, Darrah JM, et al. Epcoritamab SC + R-Mini-CHOP leads to high complete metabolic response rates in patients with previously untreated diffuse large B-cell lymphoma ineligible for full-dose R-CHOP: First disclosure from arm 8 of the EPCORE NHL-2 trial. Blood. 2023;142(Supplement 1):4457.
39. Brody J, Joergensen JM, Belada D, Costello RT, Trneny M, Vitolo U, et al. Epcoritamab SC + GemOx leads to high complete metabolic response rates in patients with relapsed/refractory diffuse large B-cell lymphoma ineligible for autologous stem cell transplant: Updated results from Epcore NHL-2. Blood. 2023;142(Supplement 1):3092.
40. Abrisqueta P, Falchi L, Phillips TJ, De Vos S, Nijland M, Offner F, et al. Subcutaneous epcoritamab + R-DHAX/C in patients (pts) with relapsed or refractory (R/R) diffuse large B-cell lymphoma (DLBCL) eligible for autologous stem cell transplant (ASCT): Preliminary phase 1/2 results. Journal of Clinical Oncology Conference: Annual Meeting of the American Society of Clinical Oncology, ASCO. 2022;40(16 Supplement 1).
41. Thieblemont C, Phillips T, Ghesquieres H, Cheah CY, Clausen MR, Cunningham D, et al. Epcoritamab, a novel, subcutaneous CD3xCD20 bispecific T-cell-engaging antibody, in relapsed or refractory large B-cell lymphoma: Dose expansion in a phase I/II trial. Journal of Clinical Oncology. 2023;41(12):2238-47.
42. Vose JM, Feldman T, Chamuleau MED, Kim WS, Lugtenburg P, Kim TM, et al. Mitigating the risk of cytokine release syndrome (CRS): Preliminary results from a DLBCL cohort of Epcore NHL-1. Blood. 2023;142(Supplement 1):1729.
43. Patel K, Riedell PA, Tilly H, Ahmed S, Michot JM, Ghesquieres H, et al. A phase 1 study of plamotamab, an anti-CD20 x anti-CD3 bispecific antibody, in patients with relapsed/refractory non-Hodgkin's lymphoma: Recommended dose safety/efficacy update and escalation exposure-response analysis. Blood. 2022;140(Supplement 1):9470-2.
44. Song Y, Li L, Qian Z, Zhou K, Fan L, Tan P, et al. GB261, an Fc-function enabled and CD3 affinity de-tuned CD20/CD3 bispecific antibody, demonstrated a highly advantageous safety/efficacy balance in an ongoing first-in-human dose-escalation study in patients with relapsed/refractory non-Hodgkin lymphoma. Blood. 2023;142(Supplement 1):1719.
45. Olszewski AJ, Phillips TJ, Hoffmann MS, Armand P, Kim TM, Yoon DH, et al. Mosunetuzumab in combination with CHOP in previously untreated DLBCL: safety and efficacy results from a phase 2 study. Blood Advances. 2023;7(20):6055-65.
46. Matasar M, Bartlett NL, Shadman M, Budde LE, Flinn I, Gregory GP, et al. Mosunetuzumab safety profile in patients with relapsed/refractory B-cell non-Hodgkin lymphoma: Clinical management experience from a pivotal phase I/II trial. Clinical Lymphoma, Myeloma & Leukemia. 2024;24(4):240-53.
47. Budde LE, Olszewski AJ, Assouline S, Lossos IS, Diefenbach C, Kamdar M, et al. Mosunetuzumab with polatuzumab vedotin in relapsed or refractory aggressive large B cell lymphoma: a phase 1b/2 trial. Nature Medicine. 2024;30(1):229-39.
52. Budde LE, Assouline S, Sehn LH, Schuster SJ, Yoon SS, Yoon DH, et al. Single-agent mosunetuzumab shows durable complete responses in patients with relapsed or refractory B-cell lymphomas: Phase I dose-escalation study. Journal of Clinical Oncology. 2022;40(5):481-91.
